# Anti-inflammatory, anti-oxidant effect and cytotoxicity of ocimum sanctum intra oral gel for combating periodontal diseases

**DOI:** 10.6026/973206300161026

**Published:** 2020-12-31

**Authors:** Jaiganesh Ramamurthy, Nadathur Duraisamy Jayakumar

**Affiliations:** 1 Department of Periodontics, Saveetha Dental College and Hospitals, Saveetha Institute of Medical and Technical Sciences (SIMATS), Saveetha University, Chennai, India

**Keywords:** Ocimum sanctum, periodontal diseases, anti-oxidant effect, anti-inflammatory effect, cytotoxic effect

## Abstract

It is of interest to evaluate the anti-inflammatory, anti-oxidant effect and cytotoxicity of Ocimum sanctum (an Indian herb, Thulsi) intra oral gel in combating periodontal diseases. Hence, 2% of O. sanctum gel was prepared with Carbopol940 soaked in purified water containing 0.2% w/v sodium benzoate overnight. Hydroxy proplyl methyl cellulose (HPMC) solution was mixed with propylene glycol using using tissue homogenizer. Anti-oxidant effect was analyzed using DPPH radical assay and anti-inflammatory effect was assessed using the inhibition of albumin denaturation assay. Ocimum sanctum gel with various dilutions from10 micro litres to 50 micro litres showed exponential increase in percentage of inhibition from 60.9 to 72.2 exhibiting antioxidant activity. The anti-inflammatory effect of Ocimum sanctum gel showed comparatively equivalent effect with standard diclofenac gel with values ranging from 76.6 for 50 micro liters of Ocimum sanctum gel and 89.6 for standard gel at 50 micro liters. Ocimum sanctum showed less toxicity towards brine shrimp nauplii. Thus we show that Ocimum sanctum gel showed potent anti-oxidant and anti-inflammatory effect and less toxic to brine shrimp nauplii as a promising agent for the treatment of periodontal diseases.

## Background

Periodontal diseases are chronic inflammatory diseases in which host, microbes, environment and genetics plays an important role in causing the disease or alter the rate of progression of the disease. Dental plaque, which exists in a state of biofilm, is the etiological agent for periodontal disease [1]. The organisms, which are commonly detected in Periodontitis, are Porphyromonas gingivalis, Aggregatibacter actinomycetem comitans, Tannerella forsythia, Prevotella intermedia. These microbes are present in sub gingival dental plaque and exist in a state of biofilm. In the presence of biofilm, antimicrobial agents won't be effective in killing the organisms. Hence it's very important to destroy the biofilm environment for controlling the organisms [2]. Treatment of periodontal disease aims at removal of etiological factors like plaque and calculus by scaling and root planing there by removing the biofilm loaded with microrganisms. But certain microorganisms like A.a, P.g reside inside the tissues and also in echoniches and repopulate the diseased site [3]. These microorganisms trigger the host inflammatory process and make it as hyper inflammatory trait, leading to release of enzymes like Matrix Metalo Proteinases (MMP) to host tissues. The destruction from the hyperresponsive host inflammation is more than the microorganism itself. Hence modulating the host immune response holds a paramount importance. Anti-inflammatory agents, anti-oxidants to control reactive oxygen species, anti-cytokine therapy have been tried for controlling the host inflammatory response. All these therapies has unwanted side effects when used in long term, hence search for natural extract which has anti-microbial, anti-inflammatory, and anti-oxidant property would be beneficial for the treatment of periodontal disease [4,5]. Ocimum sanctum (O.sanctum) is known as the "Queen of Herbs". It is described as sacred and medicinal plant in ancient literature and has been used in different formulations for the treatment of a wide range of disorders, including those of the mouth and throat, lungs, heart, blood, liver, kidney and other systems. It is also called Holy Basil and in Sanskrit Tulsi means 'the incomparable one’ has got two varieties, Krishna Tulsi (black) and Rama Tulsi (green). O. sanctum has demonstrated anti-microbial, anti-oxidant and anti-inflammatory effects in various pharmacological studies [6]. Therefore, the aim of the study was to estimate the anti-inflammatory and anti-oxidant effect of Ocimum sanctum intra oral gel for the treatment of periodontal disease.

## Methodology

The study was presented to institutional ethical committee and got approved. The study was in accordance with Helsinki's declaration. There are three parts in materials and methods. First is preparation of Ocimum sanctum gel followed by evaluation of antiinflammatory activity and evaluation of anti-oxidant activity of the prepared Ocimum sanctum insitu gel.

## Preparation of Ocimum sanctum gel:

Ethanolic extract of O. sanctum was prepared by maceration method. 250gm of Tulsi powder is soaked in 1000 ml of ethyl alcohol for 48hrs and filtered with whatman filter paper. Filtered liquid was evaporated to get supercritical fluid and stored in suitable container with appropriate temperature until further use. Two percentage of O. sanctum gel was prepared with following method: Carbopol940 was soaked in purified water containing 0.2% w/v sodium benzoate overnight. Using tissue homogenizer hydroxyproplyl methyl cellulose (HPMC) solution was mixed with propylene glycol. 2ml of tulsi extract (supercritical fluid extract) was transferred into HPMC solution and homogenized (see PDF Figure 1). This drug solution were later transferred to carbapol solution and homogenized. Triethanolamine was added to neutralize the pH. (Figure 1 - see PDF)

## Antioxidant Activity:

DPPH radical assay: The DPPH free radical scavenging activity of Ocimum sanctum gel was determined according to the methodology followed in this study [7]. Typically, different concentration (2-10 µg/ml) of plant extract was mixed with 1 ml of 0.1 mM DPPH in methanol solution and 450 µl of 50 mM Tris-HCl buffer (pH 7.4) and incubated for 30 min. After incubation, the reduction in the number of DPPH free radicals was measured based on the absorbance at 517 nm. BHT was used as controls. (Figure 2 and Figure 3 - see PDF) The percent inhibition was calculated from the following equation: % Inhibition = [Absorbance of control -Absorbance of test sample/Absorbance of control] x 100 results are shown in (Table 1 & Figure 5 - see PDF)

## Anti-inflammatory activity:

Inhibition of albumin denaturation assay: BSA (Bovine serum albumin) was used as a reagent for the assay. Bovine serum albumin (BSA) makes up approximately 60% of all proteins in animal serum. It is commonly used in cell culture, particularly when protein supplementation is necessary and the other components of serum are unwanted. BSA undergoes denaturation upon heating and starts expressing antigens associated with Type III hypersensitive reaction which are related to diseases such as rheumatoid arthritis, glomerulonephritis, serum sickness and systemic lupus erythematosus. 2 mL of 1% Bovine albumin fraction was mixed with 400 µL of plant crude extract in different concentrations (500-100 µg/mL) and the pH of reaction mixture was adjusted to 6.8 using 1N HCl. The reaction mixture was incubated at room temperature for 20 minutes and then heated at 55°C for 20 min in a water bath. The mixture was cooled to room temperature and the absorbance value was recorded at 660 nm. An equal amount of plant extract was replaced with DMSO for control. Diclofenac sodium in different concentrations was used as standard [8]. The experiment was performed in triplicate. (Table 2 & Figure 6 - see PDF)

% Inhibition was calculated using the following formulae:

% Inhibition = Control O.D- sample O.D Control O.D * 100

## Cytotoxicity Test:

Cytotoxic Assessment was done by brine shrimp egg. In this experiment, brine shrimp eggs are kept in the fish tank and iodide free salt is added in the beaker along with water. The solution is then stirred for 10 minutes till the crystallized salt gets dissolved. Sodium bicarbonate was then added in the fish tank in small amount of 0.5mg. The brine shrimp eggs were kept in the tank for24 hours. After 24 hours nauplii were hatched and they were identified and separated. Then 10 naupliis are added in each Elisa well and O. sanctum insitu gel was added in the range of 10µl, 20µl, 30µl, 40µl, 50µl in all wells and control level also added. To determine the mortality rate of prepared O. sanctum insitu gel movement of nauplii was noted and stable ones were checked under microscope to assess it micromovement (Table 3 - see PDF).

## Results and Discussion:

Treatment of periodontal disease starts at removing the etiological factors and disturbing the biofilm. Anti-microbial therapy is given to reduce the microbes penetrated inside the tissues and eradicate the organisms from econiche. It can be done systemically as well as locally. Systemic medications has side effects hence locally delivered drugs into gingival crevice has the advantage. Locally delivered drugs should be retained in the sulcus for required period of time and it should be above the minimum inhibitory concentration for periodontal pathogens. Pharmacokinetics of the locally delivered drugs should follow zero order kinetics for effective drug delivery. Drugs like tetracycline, doxycycline, minocycline, metronidazole, chlorhexidine has been tried in the treatment of periodontal disease with potential effects. But these drugs don't have anti-inflammatory, anti-oxidant property to modulate the host. Hence search of drug or natural extract, which has antimicrobial, antioxidant and anti-inflammatory property, is in place [9]. O. sanctum (Tulsi) belongs to plant family Lamiaceae. It has made important contribution to the field of science from ancient times as also to modern research due to its large number of medicinal properties. Phytoconstituents of Tulsi: The leaves of O. sanctum contain 0.7% volatile oil comprising about 71% eugenol and 20% methyl eugenol. The oil also contains carvacrol and sesquiterpine hydrocarbon caryophyllene 4. Fresh leaves and stem of O. sanctum extract yielded some phenolic compounds (antioxidants) such as cirsilineol, circimaritin, isothymusin, apigenin and rosameric acid, and appreciable quantities of eugenol [10]. Two flavonoids, viz., orientin and vicenin from aqueous leaf extract of O. sanctum have been isolated. Ursolic acid, apigenin, luteolin, apigenin-7-O-glucuronide, luteolin-7-O glucuronide, orientin and molludistin have also been isolated from the leaf extract6. O. sanctum also contains a number of sesquiterpenes and monoterpenes viz., bornyl acetate, elemene, neral, and pinenes, camphene, campesterol, cholesterol, stigmasterol and sitosterol [11]. A study done by Vishwabhan et al. proposed the antimicrobial activity of Tulsi by virtue of its essential oil content [12]. Various other studies also project the idea that Tulsi yields various essential oils, which are responsible for the medicinal uses including antimicrobial, antioxidant, antifungal, and anti‑inflammatory activities that can probably explain its activity against the microbes discussed so far. It has also been postulated that Tulsi has an immunomodulatory effect and acts by increasing the levels of interferon, interleukin‑4 and T helper cells that can strengthen host response to infections [13, 14]. The methanol extract and the aqueous extract of O. sanctum have shown to inhibit acute as well as chronic inflammation in rats. Eugenol (l-hydroxy-2-methoxy-4-allylbenzene), the active constituent present in O.sanctum, has been found to be largely responsible for the anti-inflammatory property of tulsi. It demonstrated 97% cyclooxygenase-1 inhibitory activity when assayed at 1000 µM concentration [15-18]. In an animal study done by Hosadurga et al. 2% O. sanctum was used in the treatment of experimental periodontitis in Wistar albino rats. Anti-Inflammatory effect and duration of action was assessed for edema reduction. Results of the study showed O. sanctum gel showed antiinflammatory effect by reducing edema to 33.66% compared to control group and action was sustained till 24 hrs. However antioxidant effect and toxicity levels were not assessed in that study [19]. In our present study O. sanctum showed excelled anti-oxidant effect of 72.2 percentage of inhibition in 50 micro liters, which is equivalent to any standard anti-oxidant agent. Also antiinflammatory effect of O. sanctum in our study showed 76.6 percentage of inhibition in comparison with the standard diclofenac gel, which showed 89.6 percentage of inhibition at 50 microliters. Though the anti-inflammatory effect is lesser than standard drug it is sufficient for producing anti-inflammatory effect when delivered in local delivery form. Cytotoxicity of O. sanctum gel was assessed by Brine shrimp eggs assessment method. Nauplli was hatched from brine shrimp eggs and was tested with O. sanctum gel. Results showed survival rate of nauplii at variable concentrations of O. sanctum gel. Nauplii survived better after 24 hrs and 48 hrs in lesser concentrations of the gel when compared to higher concentration of the gel. Survival rate of nauplii is within the acceptable limits.

## Conclusion

Ocimum sanctum has shown promising actions towards the treatment of periodontal diseases by having anti-inflamatory, antioxidinat action with less toxicity. Cytoxicity test showed O. sanctum insitu gel is less toxic. O. sanctum gel showed potent anti-oxidant and anti-inflammatory effect comparable to standard drug. O. sanctum intra oral gel is a potential local drug delivery agent in the treatment of periodontal disease. However, adequate clinical trials are required to validate its therapeutic potential.
